# Supplementary material for: A NAC-EXPANSIN module enhances maize kernel size by controlling nucellus elimination
Source: Nat Commun. 2022 Sep 29;13:5708. doi: 10.1038/s41467-022-33513-4 (PMC9522829; doi:10.1038/s41467-022-33513-4)
Supplement: Supplementary file 1 — Supplementary information [file 41467_2022_33513_MOESM1_ESM.pdf]

A NAC-EXPANSIN module enhances maize kernel size by controlling nucellus elimination

Sun *et al*.

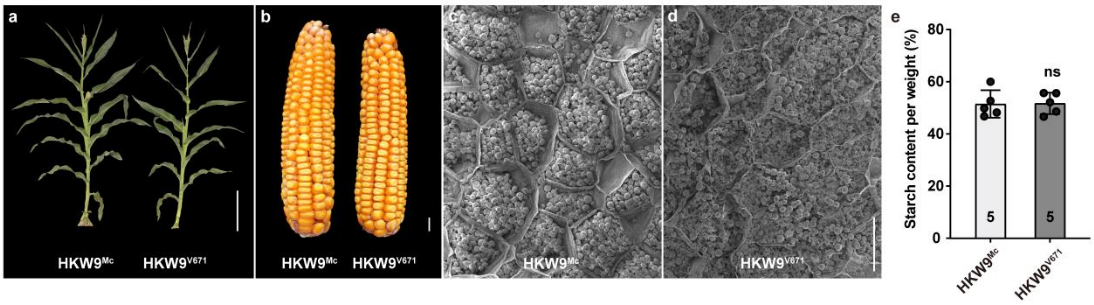

Supplementary Fig. 1. Mature plant and ear architectures and starch accumulation of HKW9^{Mc} and HKW9^{V671}. a, b, Mature plant (**a**) and ear architectures (**b**) of HKW9^{Mc} and HKW9^{V671} are indistinguishable. **c, d,** The endosperm cells of HKW9^{Mc} (**c**) and HKW9^{V671} (**d**) kernels at the filling stage of 18 days after pollination (DAP). **e,** Total starch content in mature kernels of HKW9^{Mc} and HKW9^{V671}. Scale bar = 30 cm in (**a**), 1 cm in (**b**) and 100 μm in (**c, d**). Values in (**e**) are means ± s.d. (standard deviation, n = 5 independent samples), and the significance is estimated by the one-way ANOVA. ns, non-significance. Source data are provided as a Source Data file.

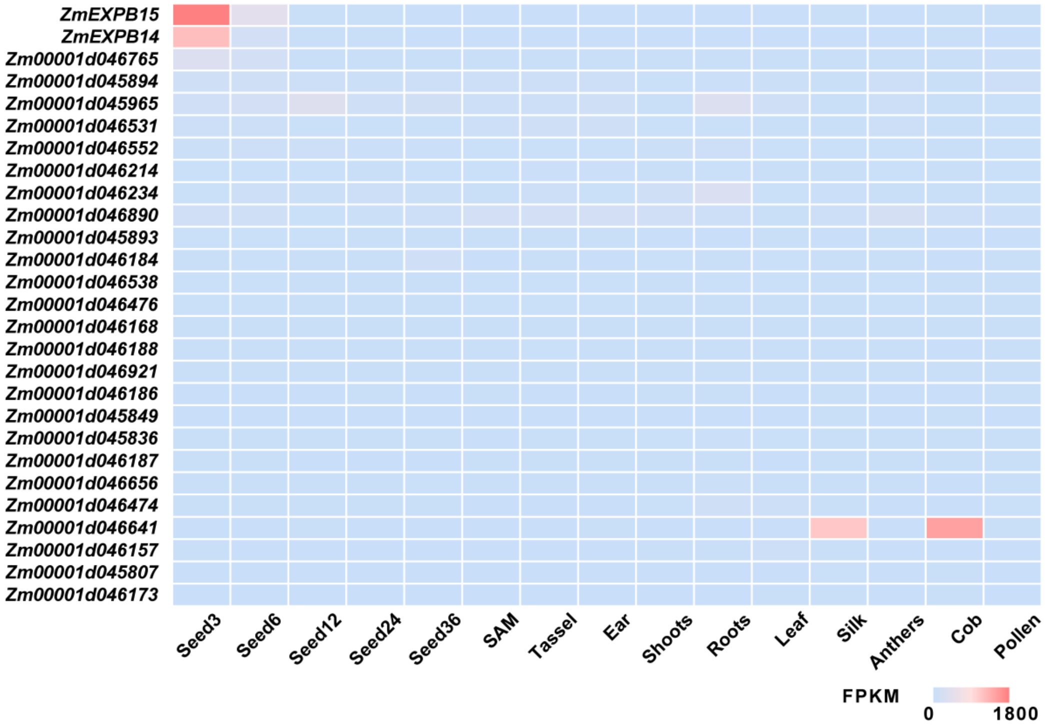

Supplementary Fig. 2. Expression profiles of the 27 annotated genes from the candidate mapping region based on the public RNA-seq data[1]. The tissues are indicated at the bottom. Seed3-Seed36: developing seeds from 0 to 36 days after pollination (DAP)[2]. SAM, shoot apices (pools of 15) dissected were from 19 days old plants showing 6-7 leaves. Tassel, tassels (pools of 10) dissected were 4-5.5 mm. Ear, ears (pools of 6-8) dissected were 7-8 mm[3]. Shoots and Roots, harvested seedlings after 14 days, separated into shoots and roots[4]. Leaf, pooled leaves at 20 after sowing. Anthers, whole anther including pollen dissected from tassel structural tissue at anthesis. Cob, immature cob at 10 days before cob emergence. Pollen, mature pollen at anthesis. Silk, mature silk at anthesis[5]. All tissue materials were collected from B73 inbred line. The color bars represent the expression values (FPKM, fragments per kilobase of transcript per million mapped reads). Source data are provided as a Source Data file.

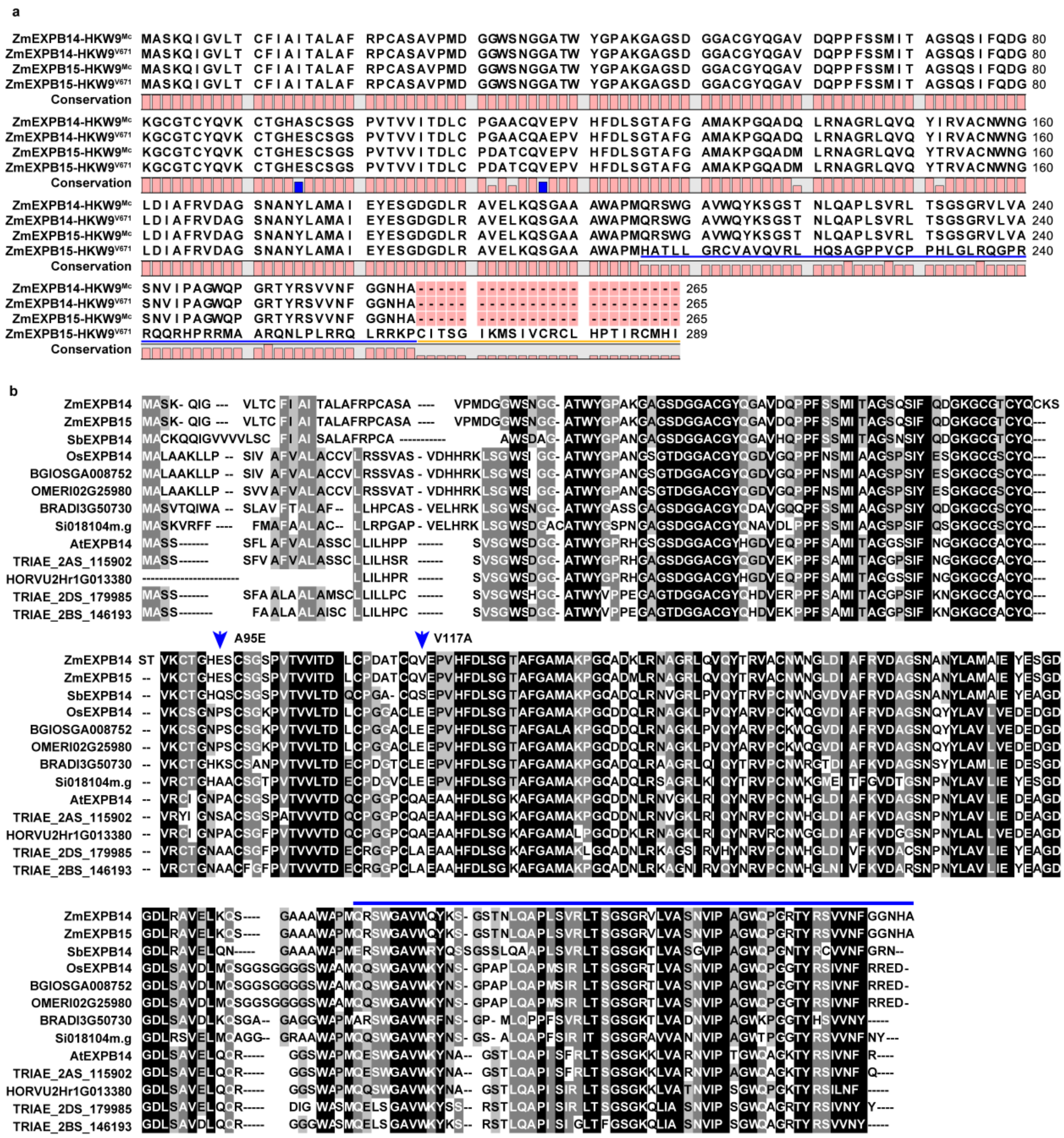

Supplementary Fig. 3. Alignment of the amino acid sequences of ZmEXPB14 and ZmEXPB15, and the homologues in other species. a, Alignment of the amino acid sequences of ZmEXPB14 and ZmEXPB15 in HKW9^{Mc} and HKW9^{V671}. *ZmEXPB14* contained two nonsymponous mutation (blue box, A95E, V117A) in two NILs. *ZmEXPB15* covered a 4-bp deletion in the last coding exon, leading to a frame shift of the last 60 amino acids (blue underline) and an addition of 24 residues (yellow underline) in the C-terminus. **b,** Alignment of the amino acid sequences of ZmEXPB14 and ZmEXPB15, and their homologues in other species. Black shaded amino acids represent the residues with 80% identity and gray ones indicate the similar residues. *ZmEXPB14* contained two nonsymponous mutations in the non-conserved (blue arrows, A95E, V117A). *ZmEXPB15* contained a frame shift mutation of the last 60 amino acids in the conserved domain (blue line).

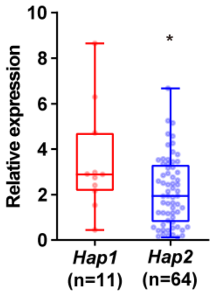

Supplementary Fig. 4. Box-and-whisker plots of *ZmEXPB15* expression among 75 diverse inbred lines of the *Hap1* (red box, n=11) and *Hap2* (blue box, n=64). The significance is estimated using a one-way ANOVA. *P < 0.05. One biological replicate and three technical replicates for each line. n is the number of inbred lines examined. Each box represents the median and interquartile range, and whiskers extend to maximum and minimum values. Source data are provided as a Source Data file.

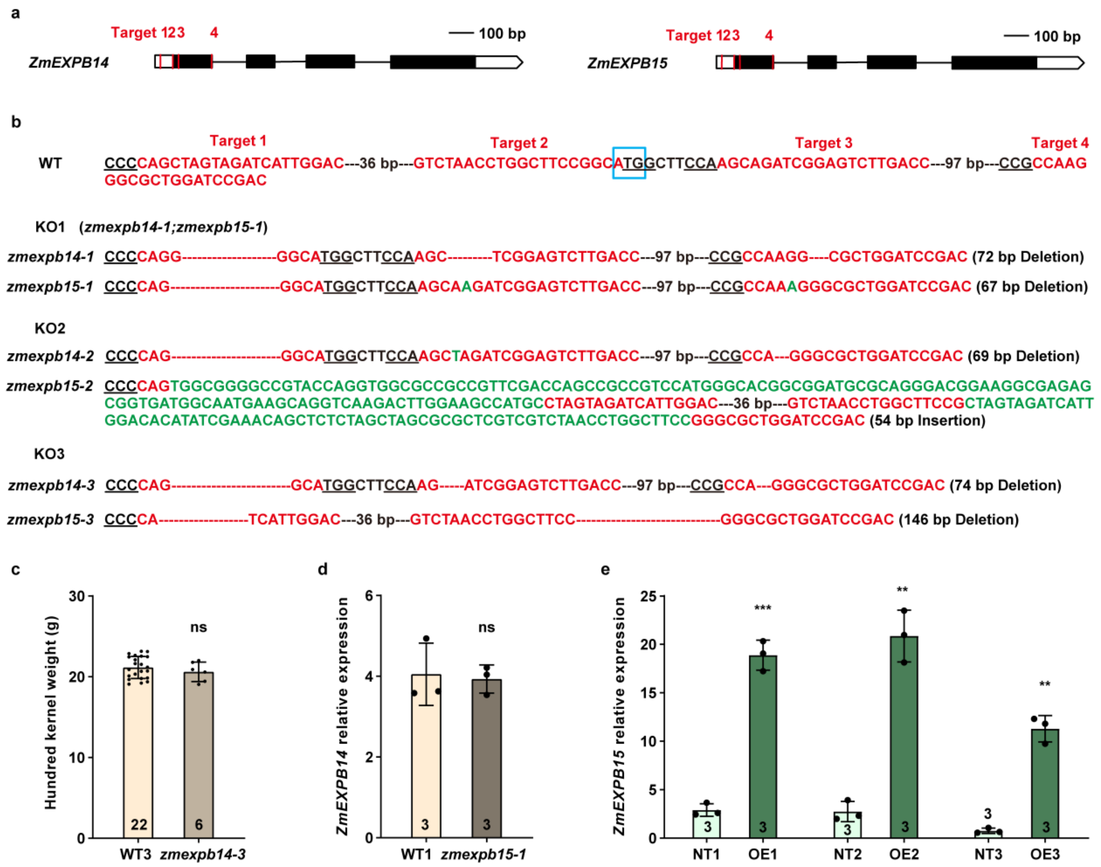

Supplementary Fig. 5. Characterization of the CRISPR-Cas9 edited *zmexpb14* and *zmexpb15* mutants. a, The gene structure of *ZmEXPB14* and *ZmEXPB15* and positions of four sgRNA targets (red lines) for CRISPR-Cas9 editing. Black rectangles indicate the exons of *ZmEXPB14* and *ZmEXPB15*. **b,** The detailed sequence edits for *ZmEXPB14* and *ZmEXPB15* in three double knockout (KO) lines. The four sgRNA target sequences are marked in red with the protospacer-adjacent motif (PAM) sites underlined, and the start codon is marked with a blue box. The insertion sequences are in green and the deletions are indicated by the red dashes. **c,** Quantitative analysis showed that *zmexpb14-3* single mutant had no change in HKW. The number on each column is the independent ears. **d,** The expression levels of *ZmEXPB14* was not altered in *zmexpb15-1* single mutant lines. All expression levels from three biological repeats were normalized to *Actin*. **e,** The expression levels of *ZmEXPB15* in three overexpression (OE1, OE2 and OE3) lines. All expression levels from three biological repeats were normalized to *Actin*. Values in (**c-e**) are means ± s.d., and the significance in (**c**) is estimated by one-way ANOVA. and the significance in (**d, e**) is estimated using a two-tailed Student's *t* test. $^{**}P < 0.01$, $^{***}P < 0.001$, ns, non-significance. Source data are provided as a Source Data file.

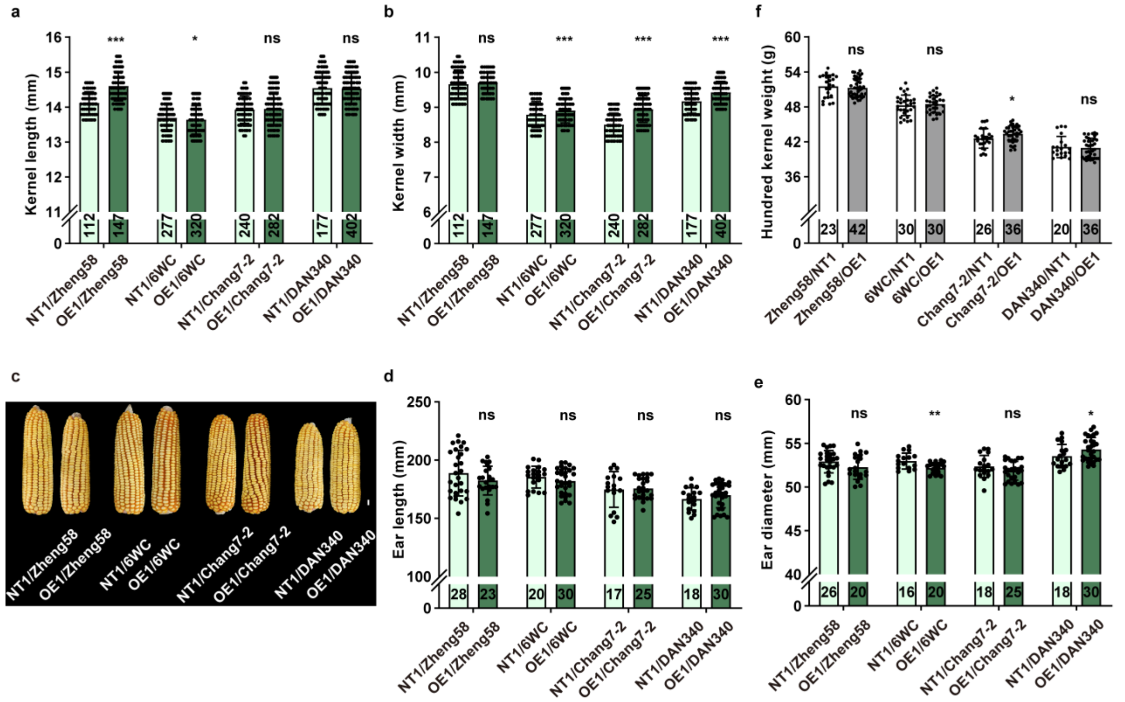

Supplementary Fig. 6. The evaluation of the ear and kernel traits of four hybrids developed from the overexpressed line (OE1). a, b, Scatter plots of kernel length (**a**) and kernel width (**b**) in the hybrids developed from the overexpression line (OE1) and the corresponding non-transgenic line (NT1), respectively, crossing to four elite inbred lines (Zheng58, 6WC, Chang7-2, Dan340). **c,** Comparison of ears status of the hybrids. Scale bar = 1 cm. **d, e,** Scatter plots of ear length (**d**) and ear diameter (**e**) of the hybrids. **f,** Scatter plots of hundred kernel weight in the hybrids developed from four inbred lines (Zheng58, 6WC, Chang7-2, Dan340) crossing to overexpression line (OE1) and the corresponding non-transgenic line (NT1), respectively. The number on each column corresponds to the sample size. Values are means ± s.d., and the significance is estimated by the one-way ANOVA. $*P < 0.05$, $**P < 0.01$, $***P < 0.001$, ns, non-significance. Source data are provided as a Source Data file.

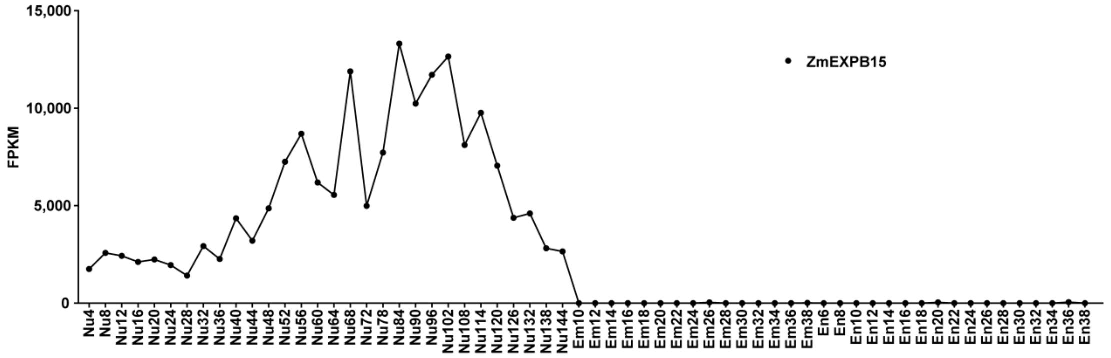

Supplementary Fig. 7. *ZmEXPB15* is highly and specifically expressed in nucellus of developing kernel. The expression profile of *ZmEXPB15* in three components of kernel is based on the public RNA-seq data[1]. Nu4-Nu144: developing nucellus from 4 to 144 HAP (hours after pollination); Em10-Em38: developing embryos from 10 to 38 DAP; En6-En38: developing endosperms from 6 to 38 DAP. Source data are provided as a Source Data file.

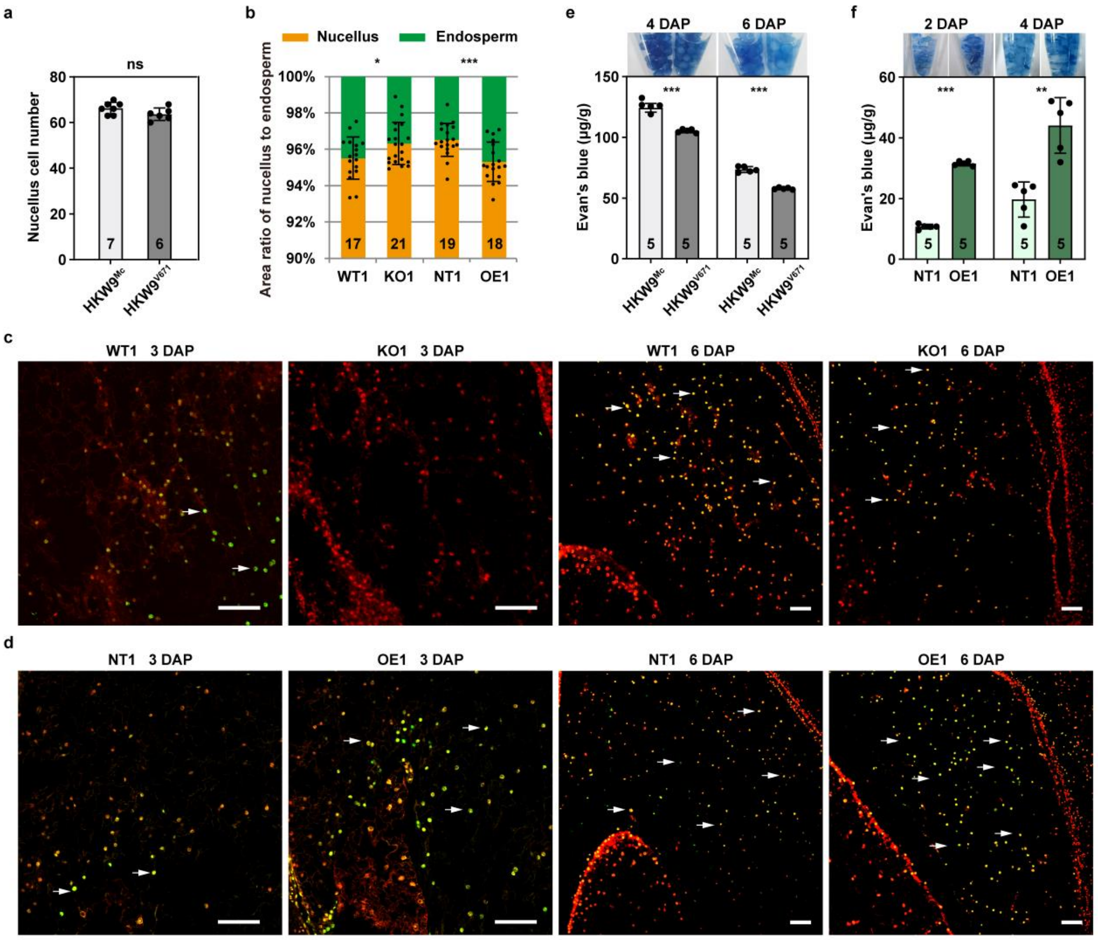

Supplementary Fig. 8. *ZmEXPB15* promotes nucellus cell elimination. a, Quantification of nucellus cell number of the largest longitudinal semi-thin section of 3-DAP kernel in two NILs. The nucellar cell number is measured along the black dotted line in Fig. 4a. **b,** Ratio of nucellus (orange box) to endosperm area (green box) of 3-DAP kernels in *ZmEXPB15* knockout line (KO1) in comparison to the wild type (WT1) control in a C01 inbred background, and overexpression line (OE1) in comparison to the non-transgenic line (NT1) in a B104 inbred background. Longitudinal median sections with maximum area of the endosperm for each line were examined and quantified. **c, d,** TUNEL assays of the nucellus at 3 and 6 DAP of KO1 in comparison to WT1 (**c**), and the OE1 in comparison to NT1 control (**d**). **e, f,** Evan's blue staining of young kernels of NILs HKW9^{Mc} and HKW9^{V671} at 4 and 6 DAP (**e**), and the kernels of OE1 in comparison to NT1 control at 2 and 4 DAP (**f**). The quantification was performed by measuring the blue dye absorbance at 600 nm and repeated five times. Number on the bottom of each column is the sample size. Values in (**a, b, e, f**) are means ± s.d., and the significance is estimated by a two-tailed Student's *t* test, $*P < 0.05$, $***P < 0.001$, ns, non-significance. Scale bar = 100 μm in (**c, d**). Source data are provided as a Source Data file.

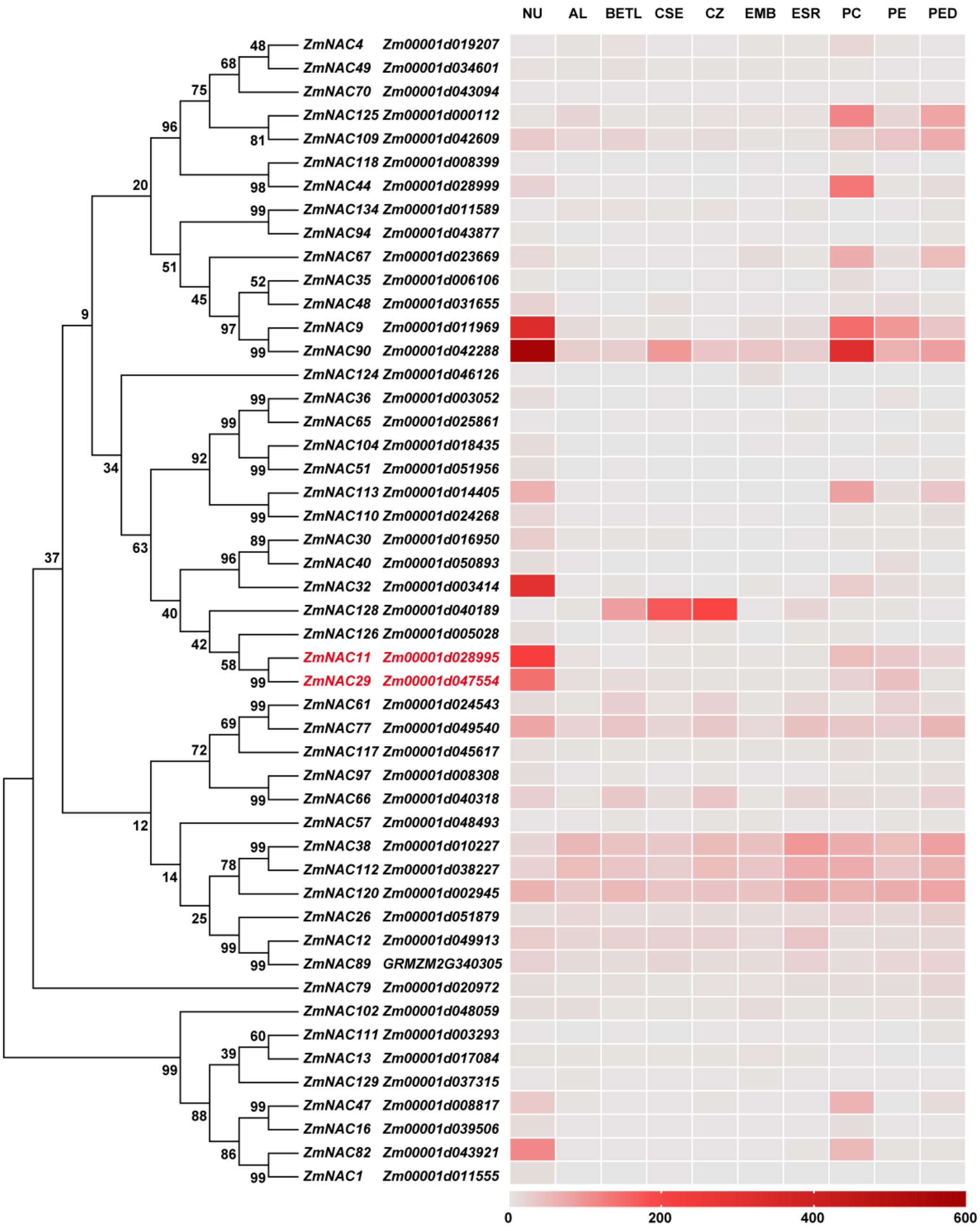

Supplementary Fig. 9. Phylogenetic analysis and expression pattern of the maize NAC TFs in developing kernel. The phylogenetic tree is produced by MEGA7.0 software based on the NAC TF family of 49 NAC proteins from Zhan et al[6]. Protein sequences are downloaded from the Gramene (http://www.gramene.org/). The 49 NAC proteins were obtained from 83 NAC transcription factor families annotated by Zhan et al. by screening the genes expressed in the 8-DAP kernel nucellar tissues. The bottom colored bar represents the expression values. The tissues are indicated at the top of each column. NU, nucellus; AL, aleurone; BETL, basal endosperm transfer layer; CSE, central starchy endosperm; CZ, conducting zone; EMB, embryo; ESR, embryo-surrounding region; PC, placento-chalazal region; PE, pericarp; PED, pedicel. Source data are provided as a Source Data file.

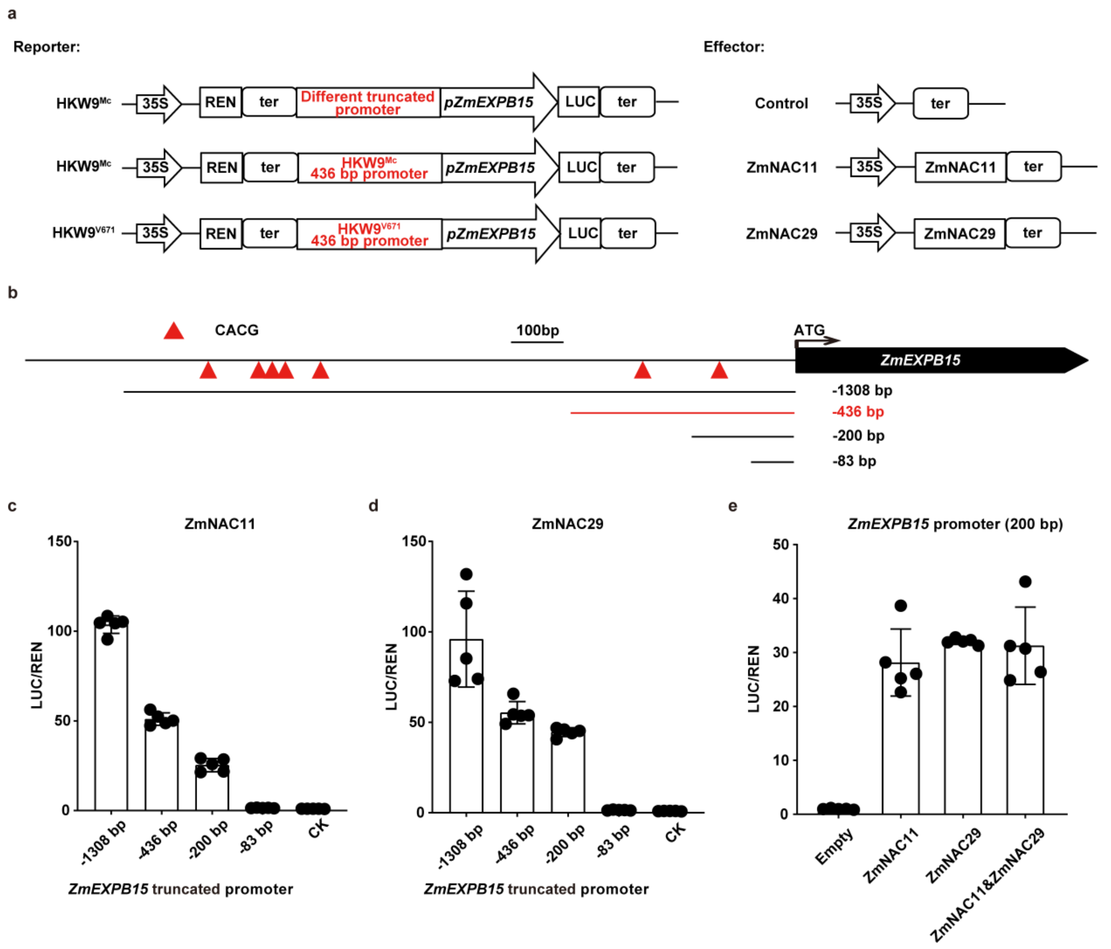

Supplementary Fig. 10. Transactivation effects ZmNAC11 and ZmNAC29 on *ZmEXPB15*. a, Schematic diagrams of the reporter and effector constructs. REN, Renilla luciferase; LUC, firefly luciferase; ter, terminator. **b,** The different truncated promoter of *ZmEXPB15* used for the transactivation assays using maize leaf protoplast. The numbers are the lengths of the truncations relative to ATG, and the red triangles represent the CACG motifs. **c,d,** Transactivation effects of ZmNAC11 (**c**) and ZmNAC29 (**d**) on different truncated promoters of *ZmEXPB15*. CK represents the control without a promoter fragment inserted. **e,** Relative LUC activities driven by the different promoters of 200 bp of *ZmEXPB15* transactivated by either individual protein ZmNAC11 and ZmNAC29, or combination of two NAC proteins. Empty represents the control without a protein in the assay. The charts represent mean ± s.d. of the LUC/REN ratio from five replicates. Source data are provided as a Source Data file.

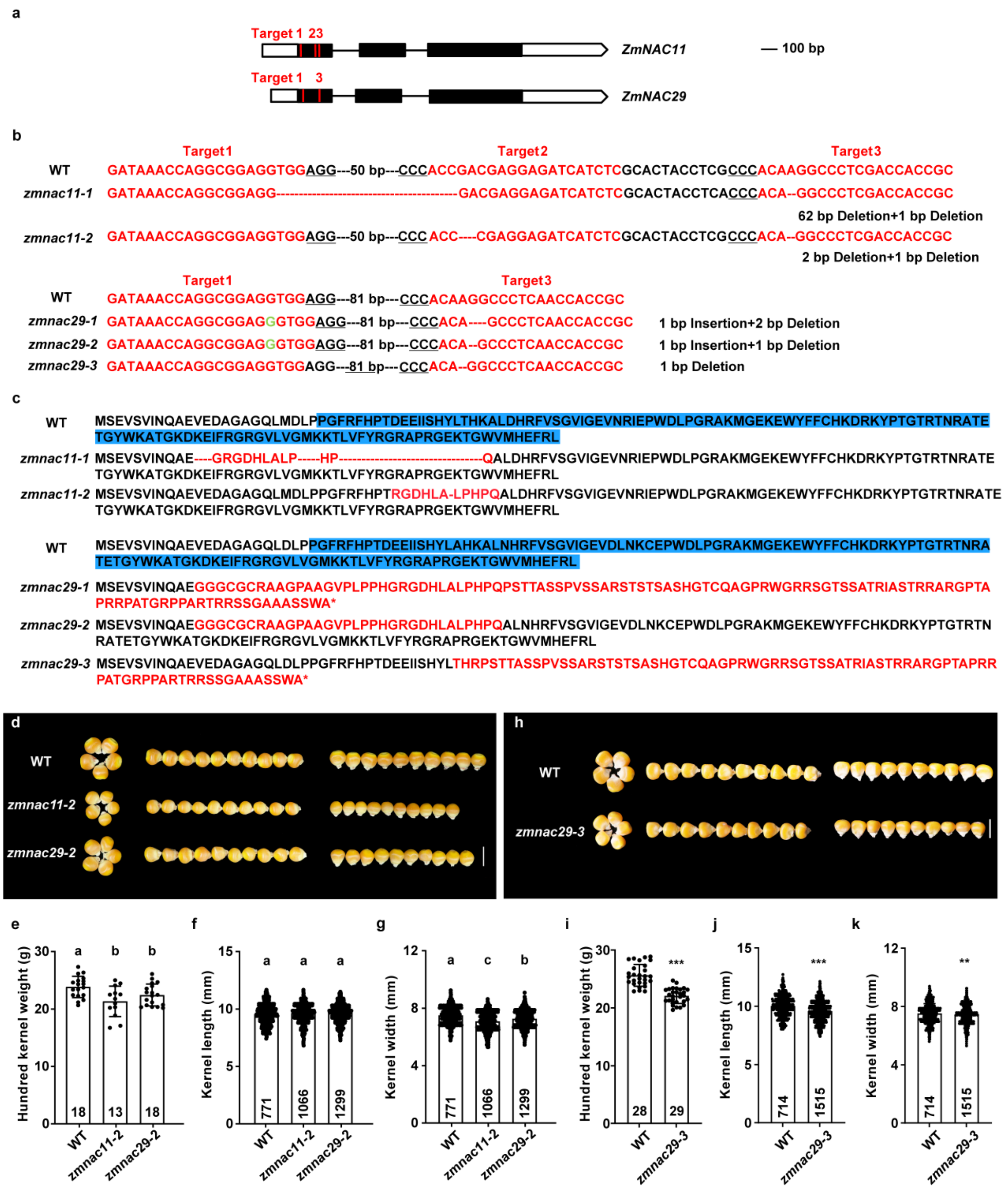

Supplementary Fig. 11. Characterization of the CRISPR-Cas9 edited *zmnac11* and *zmnac29* mutants. a, The gene structure of *ZmNAC11* and *ZmNAC29* and positions of the sgRNA targets for CRISPR-Cas9 editing. Black rectangles indicate exons. The target sites of sgRNAs are marked red. **b, c,** DNA sequences (**b**) and putative amino acid sequences (**c**) of independent transgenics of *nac* mutants. The edited sequences of each alleles are listed with the sgRNA sequences in red and protospacer-adjacent motif (PAM) sites underlined. The deletions are indicated by red dashes, and insertions are marked in green. These deletions and/or insertions lead to severe interruptions or deletions of the conserved NAC domain. Blue rectangles indicate NAC domain. Asterisks indicate a stop codon. WT, wild-type. **d,** The edited lines, *zmnac11-2* and *zmnac29-2*, develop smaller kernels than the corresponding

wild-type plants in C01 inbred background. **e-g,** Quantitative analysis of hundred kernel weight (**e**), kernel length (**f**) and kernel width (**g**) in WT, *zmnac11-2* and *zmnac29-2*. **h,** The *zmnac29-3* edited line develop smaller kernels than the corresponding wild-type plants in C01 inbred background. **i-k,** Quantitative analysis of hundred kernel weight (**i**), kernel length (**j**) and kernel width (**k**) in WT and *zmnac29-3*. Number on each column is the sample size. Scale bar = 1 cm in (**d, h**). Values in (**e-g**) are means ± s.d. and Tukey HSD test is used and statistical differences ($P < 0.05$, one-sided) are indicated by different letters. Values in (**i-k**) are means ± s.d., and the significance is estimated by the one-way ANOVA. **$P < 0.01$, ***$P < 0.001$. Source data are provided as a Source Data file.

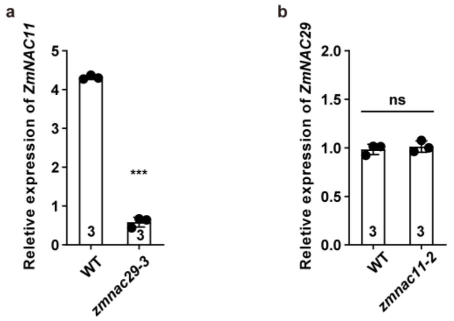

Supplementary Fig. 12. The expression of *ZmNAC11* and *ZmNAC29* in their null mutants. a, qRT-PCR analysis of *ZmNAC11* in 2-DAP kernels of *zmnac29-3* mutant and the corresponding wild type. **b,** qRT-PCR analysis of *ZmNAC29* in 2-DAP kernels of *zmnac11-2* mutant and corresponding wild type. WT, wild-type. All expression levels from three biological repeats were normalized to *Actin*. Values in (**a, b**) are estimated using a two-tailed Student's *t* test. ***$P < 0.001$. ns, non-significance. Source data are provided as a Source Data file.

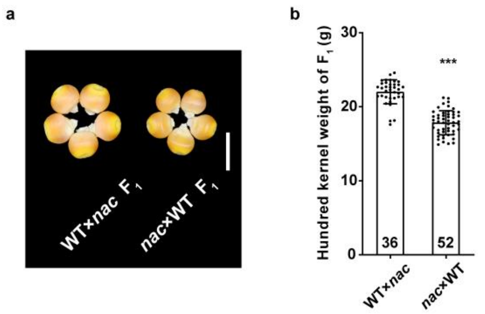

Supplementary Fig. 13. The *ZmNAC11* and *ZmNAC29* act maternally to control kernel size. a, The F_1 kernels of wild-type (WT) pollinated by *zmnac11-1*;*zmnac29-1* double mutant (*nac*) was obviously larger than those of *zmnac11-1*;*zmnac29-1* pollinated by WT. Scale bar = 1 cm. **b,** Quantitative analysis of the hundred kernel weight of F_1 kernels from the WT and *nac* reciprocal crosses. Value is means ± s.d., and the significance is estimated by one-way ANOVA. ***$P < 0.001$. Numbers on the bottom of each column are the sample size. Source data are provided as a Source Data file.

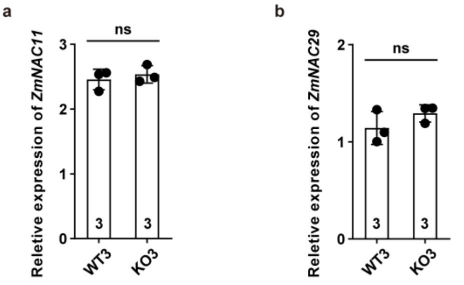

Supplementary Fig. 14. The expression of *ZmNAC11* and *ZmNAC29* were not affected by the mutation in *ZmEXPB15*. a, b, qRT-PCR analysis of *ZmNAC11* (**a**) and *ZmNAC29* (**b**) in 7-DAP kernels of *zmexpb15* mutant (KO3) and the corresponding wild type (WT3). All expression levels from three biological repeats were normalized to *Actin*. The values in (**a, b**) are estimated using a two-tailed Student's *t* test. ns, non-significance. Source data are provided as a Source Data file.

Supplementary Table 1. Significant associated variation in the *ZmEXPB15* promoter region after Bonferroni multiple test correction.

Variants	Allele	Physical position (Relative to the ATG, bp)	Frequency	*p*-value
SNP-740	C/T	-740	40/180	3.73E-03
SNP-540	G/A	-540	40/180	3.73E-03
SNP-482	T/A	-482	40/180	3.73E-03
SNP-286	C/A	-286	40/180	3.73E-03
SNP-283	G/A	-283	40/180	3.73E-03

p-value: one-way ANOVA.

Supplementary references

1. Yi, F. *et al*. High temporal-resolution transcriptome landscape of early maize seed development. *Plant Cell* **31**, 974-992 (2019).
2. Chen, J. *et al*. Dynamic transcriptome landscape of maize embryo and endosperm development. *Plant Physiol.* **166**, 252-64 (2014).
3. Bolduc, N. *et al*. Unraveling the KNOTTED1 regulatory network in maize meristems. *Genes Dev.* **26**, 1685-90 (2012).
4. Wang, X. *et al*. Genome-wide and organ-specific landscapes of epigenetic modifications and their relationships to mRNA and small RNA transcriptomes in maize. *Plant Cell* **21**, 1053-1069 (2009).
5. Davidson, R. M. *et al*. Utility of RNA sequencing for analysis of maize reproductive transcriptomes. *Plant Genome* **4**, 191-203 (2011).
6. Zhan, J. *et al*. RNA Sequencing of laser-capture microdissected compartments of the maize kernel identifies regulatory modules associated with endosperm cell differentiation. *Plant Cell* **27**, 513-531 (2015).
