# Supplementary material for: A NAC-EXPANSIN module enhances maize kernel size by controlling nucellus elimination
Source: Nat Commun. 2022 Sep 29;13:5708. doi: 10.1038/s41467-022-33513-4 (PMC9522829; doi:10.1038/s41467-022-33513-4)
Supplement: Supplementary file 3 — Description of Additional Supplementary Files [file 41467_2022_33513_MOESM3_ESM.pdf]

Description of Additional Supplementary Files

File name: Supplementary Data 1

Description: Twenty-seven annotated genes were differentially expressed between $HKW9^{Mc}$ and $HKW9^{V671}$ within 73-Mb mapping region.

File name: Supplementary Data 2

Description: The maize inbred lines list in this study.

File name: Supplementary Data 3

Description: Primers and probes used in this study.
