# Supplementary material for: A NAC-EXPANSIN module enhances maize kernel size by controlling nucellus elimination
Source: Nat Commun. 2022 Sep 29;13:5708. doi: 10.1038/s41467-022-33513-4 (PMC9522829; doi:10.1038/s41467-022-33513-4)
Supplement: Supplementary file 7 — Reporting Summary [file 41467_2022_33513_MOESM7_ESM.pdf]

nature portfolio

Corresponding author(s): Fazhan Qiu

Last updated by author(s): Sep 15, 2022

Reporting Summary

Nature Portfolio wishes to improve the reproducibility of the work that we publish. This form provides structure for consistency and transparency in reporting. For further information on Nature Portfolio policies, see our Editorial Policies and the Editorial Policy Checklist.

Statistics

For all statistical analyses, confirm that the following items are present in the figure legend, table legend, main text, or Methods section.

n/a	Confirmed	
☐	☒	The exact sample size (*n*) for each experimental group/condition, given as a discrete number and unit of measurement
☐	☒	A statement on whether measurements were taken from distinct samples or whether the same sample was measured repeatedly
☐	☒	The statistical test(s) used AND whether they are one- or two-sided<br>*Only common tests should be described solely by name; describe more complex techniques in the Methods section.*
☒	☐	A description of all covariates tested
☒	☐	A description of any assumptions or corrections, such as tests of normality and adjustment for multiple comparisons
☐	☒	A full description of the statistical parameters including central tendency (e.g. means) or other basic estimates (e.g. regression coefficient) AND variation (e.g. standard deviation) or associated estimates of uncertainty (e.g. confidence intervals)
☐	☒	For null hypothesis testing, the test statistic (e.g. *F*, *t*, *r*) with confidence intervals, effect sizes, degrees of freedom and *P* value noted<br>*Give P values as exact values whenever suitable.*
☒	☐	For Bayesian analysis, information on the choice of priors and Markov chain Monte Carlo settings
☒	☐	For hierarchical and complex designs, identification of the appropriate level for tests and full reporting of outcomes
☐	☒	Estimates of effect sizes (e.g. Cohen's *d*, Pearson's *r*), indicating how they were calculated

Our web collection on statistics for biologists contains articles on many of the points above.

Software and code

Policy information about availability of computer code

Data collection	For maize ear and kernel phenotype images: Nikon D7100 camera.<br>For kernel length and width:YTS-MKT, GREENPHENO<br>For in situ images: Nikon ECLIPSE DIC microscope.<br>For qRT-PCR: CFX96 connect real-time PCR detection system (Bio-Rad).<br>For RNA-Seq: Illumina Nova platform.<br>For scanning electron microscope: 6390LV; JEOL, Tokyo, Japan.<br>For confocal microscopy:Olympus FV1000.<br>For DLR assay: SpectraMax i3x.
Data analysis	The RNAseq datasets were analyzed by Trimmomatic v.0.33, programHISAT2 v.2.1.0, StringTie v.1.3.3b and HTSeq v.0.6.1.<br>Phylogenetic analysis was analyzed by ClustalX and MEGA program version 7.<br>One-way ANOVA, Two-tailed Student’s t test and Tukey HSD test were performed using Microsoft Excel 2010.<br>Box plot and histogram was produce using GraphPad Prism 8 software.<br>Cell area measurements were performed using Image J 1.50i.

For manuscripts utilizing custom algorithms or software that are central to the research but not yet described in published literature, software must be made available to editors and reviewers. We strongly encourage code deposition in a community repository (e.g. GitHub). See the Nature Portfolio guidelines for submitting code & software for further information.

March 2021

Data

Policy information about availability of data

All manuscripts must include a data availability statement. This statement should provide the following information, where applicable:

- Accession codes, unique identifiers, or web links for publicly available datasets
- A description of any restrictions on data availability
- For clinical datasets or third party data, please ensure that the statement adheres to our policy

The RNA-seq datasets are available from the National Center for Biotechnology Information, the BioProject and SRA accession numbers are PRJNA756197 and SRR15525330-SRR15525335 (https://www.ncbi.nlm.nih.gov/bioproject/PRJNA756197).

Human research participants

Policy information about studies involving human research participants and Sex and Gender in Research.

Reporting on sex and gender	N/A
Population characteristics	N/A
Recruitment	N/A
Ethics oversight	N/A

Note that full information on the approval of the study protocol must also be provided in the manuscript.

Field-specific reporting

Please select the one below that is the best fit for your research. If you are not sure, read the appropriate sections before making your selection.

☒ Life sciences ☐ Behavioural & social sciences ☐ Ecological, evolutionary & environmental sciences

For a reference copy of the document with all sections, see nature.com/documents/nr-reporting-summary-flat.pdf

Life sciences study design

All studies must disclose on these points even when the disclosure is negative.

Sample size	The sample-size and related analysis methods are described in the figure legend or method section.Sample size were determined based on published papers (Chen et al., 2022, doi.org/10.1038/s41467-022-32026-4).
Data exclusions	No data was excluded.
Replication	Experiments were independently repeated by multiple times and similar results were obtained. The information on replication has been included in all relevant figure legends.
Randomization	Samples were grown on the same condition and randomly allocated in the growth chamber. Experimental plant materials were collected randomly without any bias.
Blinding	For all experiments involving phenotyping, genotypes were randomized (see randomization). Genotyping was then performed independently of any phenotyping, and the genotype was not known during phenotyping. A randomized block design was used for the phenotyping of families with known genotypes, the distribution of each family in the field was generated randomly before planting. Genotypes and phenotypes were only matched for analysis after all genotyping and phenotyping data had been collected.

Reporting for specific materials, systems and methods

We require information from authors about some types of materials, experimental systems and methods used in many studies. Here, indicate whether each material, system or method listed is relevant to your study. If you are not sure if a list item applies to your research, read the appropriate section before selecting a response.

Materials & experimental systems

n/a	Involved in the study
☒	☐ Antibodies
☒	☐ Eukaryotic cell lines
☒	☐ Palaeontology and archaeology
☒	☐ Animals and other organisms
☒	☐ Clinical data
☒	☐ Dual use research of concern

Methods

n/a	Involved in the study
☒	☐ ChIP-seq
☒	☐ Flow cytometry
☒	☐ MRI-based neuroimaging
